# Identification of Early Requirements for Preplacodal Ectoderm and Sensory Organ Development

**DOI:** 10.1371/journal.pgen.1001133

**Published:** 2010-09-23

**Authors:** Hye-Joo Kwon, Neha Bhat, Elly M. Sweet, Robert A. Cornell, Bruce B. Riley

**Affiliations:** 1 Biology Department, Texas A&M University, College Station, Texas, United States of America; 2 Department of Anatomy and Cell Biology, University of Iowa, Iowa City, Iowa, United States of America; University of Pennsylvania School of Medicine, United States of America

## Abstract

Preplacodal ectoderm arises near the end of gastrulation as a narrow band of cells surrounding the anterior neural plate. This domain later resolves into discrete cranial placodes that, together with neural crest, produce paired sensory structures of the head. Unlike the better-characterized neural crest, little is known about early regulation of preplacodal development. Classical models of ectodermal patterning posit that preplacodal identity is specified by readout of a discrete level of Bmp signaling along a DV gradient. More recent studies indicate that Bmp-antagonists are critical for promoting preplacodal development. However, it is unclear whether Bmp-antagonists establish the proper level of Bmp signaling within a morphogen gradient or, alternatively, block Bmp altogether. To begin addressing these issues, we treated zebrafish embryos with a pharmacological inhibitor of Bmp, sometimes combined with heat shock-induction of Chordin and dominant-negative Bmp receptor, to fully block Bmp signaling at various developmental stages. We find that preplacodal development occurs in two phases with opposing Bmp requirements. Initially, Bmp is required before gastrulation to co-induce four transcription factors, Tfap2a, Tfap2c, Foxi1, and Gata3, which establish preplacodal competence throughout the nonneural ectoderm. Subsequently, Bmp must be fully blocked in late gastrulation by dorsally expressed Bmp-antagonists, together with dorsally expressed Fgf and Pdgf, to specify preplacodal identity within competent cells abutting the neural plate. Localized ventral misexpression of Fgf8 and Chordin can activate ectopic preplacodal development anywhere within the zone of competence, whereas dorsal misexpression of one or more competence factors can activate ectopic preplacodal development in the neural plate. Conversely, morpholino-knockdown of competence factors specifically ablates preplacodal development. Our work supports a relatively simple two-step model that traces regulation of preplacodal development to late blastula stage, resolves two distinct phases of Bmp dependence, and identifies the main factors required for preplacodal competence and specification.

## Author Summary

Cranial placodes, which produce sensory structures in the head, arise from a contiguous band of preplacodal ectoderm surrounding the anterior neural plate during gastrulation. Little is known about early regulation of preplacodal ectoderm, but modulation of signaling through Bone Morphogenetic Protein (Bmp) is clearly involved. Recent studies show that dorsally expressed Bmp-antagonists help establish preplacodal ectoderm, but it is not clear whether antagonists titrate Bmp to a discrete low level that actively induces preplacodal fate or, alternatively, whether Bmp must be fully blocked to permit preplacodal development. We show that in zebrafish preplacodal development occurs in distinct phases with differing Bmp requirements. Initially, Bmp is required before gastrulation to render all ventral ectoderm competent to form preplacodal tissue. We further show that four transcription factors, Foxi1, Gata3, Tfap2a, and Tfap2c, specifically mediate preplacodal competence. Once induced, these factors no longer require Bmp. Thereafter, Bmp must be fully blocked by dorsally expressed Bmp-antagonists to permit preplacodal development. In addition, dorsally expressed Fgf and/or Pdgf are also required, activating preplacodal development in competent cells abutting the neural plate. Thus, we have resolved the role of Bmp and traced the regulation of preplacodal development to pre-gastrula stage.

## Introduction

**Figure 1:**
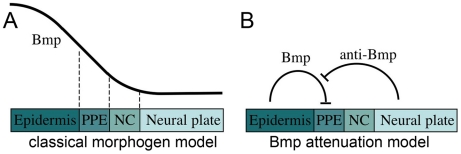
Models for the role of Bmp in preplacodal specification. (A) Classical model in which a Bmp morphogen gradient directly specifies multiple fates, including epidermal ectoderm, preplacodal ectoderm (PPE), neural crest (NC) and neural plate, at discrete threshold concentrations. (B) Bmp-attenuation model in which Bmp-antagonists, secreted from the dorsal tissue of the embryo, promotes preplacodal fate in nonneural ectoderm abutting the anterior neural plate. In this model, Bmp must be fully blocked to permit preplacodal specification.

Cranial placodes provide major contributions to the paired sensory organs of the head. Examples include the anterior pituitary, the lens of the eye, the olfactory epithelium, the inner ear, and clusters of sensory neurons in the trigeminal and epibranchial ganglia [1]–[4]. Though diverse in fate, all placodes are thought to arise from a zone of pluripotent progenitors termed the preplacodal ectoderm. Preplacodal cells arise from the nonneural ectoderm immediately adjacent to neural crest. Neural crest cells originate in the lateral edges of the neural plate and later migrate to placodal regions to contribute to the corresponding sensory structures [1], [2]. However, while neural crest has been analyzed extensively, little is known about the early requirements for preplacodal development. Various preplacodal markers, including members of the *eya*, *six* and *dlx* gene families, are expressed at high levels along the neural-nonneural interface around the anterior neural plate near the end of gastrulation [1]–[7]. How these genes are regulated is still unclear, but modulation of Bmp signaling appears to be critical. In a classical model (Fig. 1A), ectoderm is patterned during gastrulation by readout of a Bmp morphogen gradient. Such a gradient could coordinate specification of preplacodal ectoderm and neural crest in juxtaposed domains, with preplacodal ectoderm requiring slightly higher levels of Bmp than neural crest [8]–[15].

Numerous studies provide strong support for the notion that neural crest requires a specific low threshold of Bmp signaling. In zebrafish mutations or inducible transgenes that weaken overall Bmp signaling can expand neural crest throughout the ventral domain [12], [13], [15]. Similarly, development of neural crest in *Xenopus* is stimulated by misexpression of moderate but not high levels of Bmp-antagonists [11].

In contrast, available data are ambiguous with regard to Bmp's role in preplacodal specification. A number of Bmp-antagonists expressed near the neural-nonneural interface late in gastrulation are required for normal preplacodal development [16], [17]. Similarly, high-level misexpression of Bmp antagonists expands preplacodal gene expression partway into the nonneural ectoderm [18]–[21]. These findings have been alternately interpreted as support for either of two competing models: Some investigators have argued that Bmp-antagonists titrate Bmp signaling to a specific level appropriate for preplacodal specification, consistent with the Bmp morphogen model [18], [19] (Fig. 1A). Others counter that these misexpression conditions are likely to fully block Bmp signaling [20], [21], leading to an alternative model in which preplacodal specification requires attenuation of Bmp (Fig. 1B). These opposing models invoke fundamentally different mechanisms: In the morphogen model Bmp is a positive requirement whereas in the attenuation model Bmp is an inhibitor that must be fully blocked to permit preplacodal development. Notably, none of these studies has measured changes in the level of Bmp signaling associated with their experimental manipulations, making it impossible to distinguish between the opposing models. A similar uncertainty applies to genetic studies in zebrafish, which suggest that neither of the models in Fig. 1 is fully adequate. Mutations that strongly impair Bmp signaling eliminate preplacodal development [12], [13], revealing a definite requirement for Bmp. However, none of the mutations that impair Bmp to a lesser degree expand preplacodal fate throughout the ventral ectoderm, in sharp contrast to neural crest [12], [13]. Although these data fail to support predictions of the Bmp morphogen model for preplacodal specification, it is possible that available mutations do not expand the appropriate range of Bmp signaling required for preplacodal ectoderm, if one exists. Thus the status of Bmp signaling during preplacodal specification remains an important unresolved question.

In addition to differing requirements for Bmp, preplacodal ectoderm and neural crest appear to be specified at different times. Recent studies in chick and zebrafish suggest that neural crest is specified by the beginning of gastrulation [15], [22]. In contrast, preplacodal ectoderm appears to be specified during late gastrula or early neurula stages, as suggested by studies in chick and *Xenopus* [20], [21]. This difference in timing is especially relevant for the Bmp-attenuation model (Fig. 1B). Specifically, the lag in preplacodal specification allows time to reshape the Bmp gradient without jeopardizing the earlier requirement of neural crest for Bmp. There are currently no data to show when preplacodal specification occurs in zebrafish.

Other signals from dorsal tissues also appear critical for preplacodal development. In chick and *Xenopus*, grafting neurectoderm into more ventral regions induces expression of preplacodal markers in surrounding host tissue [20], [21], [23]. Moreover, combining misexpression of Bmp antagonists with Fgf8, a relevant dorsal signal, is sufficient to induce at least some preplacodal markers; neither Fgf8 nor Bmp-antagonism is sufficient [20], [21]. Various transcription factors have also been implicated in preplacodal development, but most appear to act after preplacodal specification to influence fates of cells in different regions of this domain [2], [3].

Here we provide the first direct evidence for a 2-step model in which Bmp is required only transiently during blastula/early gastrula stage to directly or indirectly induce ventral expression of four transcription factors, Tfap2a, Tfap2c, Gata3 and Foxi1, which establish preplacodal competence throughout the nonneural ectoderm. In this context, Bmp does not act as a morphogen because it does not distinguish between preplacodal and epidermal ectoderm within the nonneural domain. We initially focused on *foxi1*, *gata3*, *tfap2a* and *tfap2c* as potential competence factors because they show similar early expression patterns throughout the nonneural ectoderm and all have been implicated in later development of various subsets of cranial placodes [2], [3], [24]–[29]. Once expressed, preplacodal competence factors no longer require Bmp for their maintenance. Near the end of gastrulation, Bmp must be fully blocked by dorsally expressed Bmp-antagonists, which combined with Fgf, are necessary and sufficient to induce preplacodal development within the zone of competence.

## Results

### Requirements for Bmp

To monitor early preplacodal development, we followed expression of *dlx3b*, *eya1* and *six4.1*. *dlx3b* is the earliest marker, initially showing a low level of expression throughout the nonneural ectoderm at 8 hpf, with strong upregulation in preplacodal ectoderm and downregulation in ventral ectoderm by 9 hpf (late gastrulation) [5]. Expression of *six4.1* and *eya1* first appear in preplacodal ectoderm by 10 hpf (the close of gastrulation), and a low level of *six4.1* is also seen in scattered mesendodermal cells in the head [6], [7]. For comparison, we also monitored the neural crest marker *foxd3*, which is expressed specifically in premigratory neural crest by 10 hpf [30], [31].

To assess the role of Bmp in preplacodal specification, we treated embryos at various times with dorsomorphin (DM), a pharmacological inhibitor of Bmp signaling [32]. Although we used DM at higher concentrations than previously reported [32], it did not appear to cause defects beyond the phenotypes associated with Bmp pathway mutants (see below). Thus, unintended non-specific effects of the drug, if present, are apparently mild and do not interfere with the ability to block Bmp signaling.

We initially performed a dose-response to assess the effects of DM when added at 5, 6 or 7 hpf (Table 1). As expected, embryos were increasingly dorsalized after exposure to increasing concentrations of DM, and earlier exposure caused greater dorsalization than later exposure. Exposing embryos to 50 or 100 µM DM beginning at 5 hpf mimicked strong loss of function mutations in the Bmp pathway [8], [12], [13], [33] and resulted in complete dorsalization (Table 1). In confirmation, exposure to 100 µM DM at 5 hpf eliminated phospho-Smad1/5/8 staining within 15 minutes (Fig. S1A), indicating rapid and complete cessation of Bmp signaling. Additionally, mRNA for *sizzled*, a feedback inhibitor of Bmp [34], decayed rapidly under these conditions, with only weak staining after 30 minutes and none after 1 hour (Fig. S1B).

**Table 1:** Stage- and dose-dependent dorsalization caused by dorsomorphin (DM).

		100µM	50µM	25µM	12.5µM	6.25µM
	n	19	18	20	25	30
DM@5hpf	%C5	100	100	45	0	0
	%C4	0	0	55	0	0
	%C3	0	0	0	0	0
	%C2	0	0	0	52	0
	%C1	0	0	0	48	13
	n	25	23	19	18	18
DM@6hpf	%C5	72	22	0	0	0
	%C4	28	43	21	0	0
	%C3	0	35	79	0	0
	%C2	0	0	0	28	0
	%C1	0	0	0	72	0
	n	19	19	19	19	23
DM@7hpf	%C5	0	0	0	0	0
	%C4	11	0	0	0	0
	%C3	89	100	0	0	0
	%C2	0	0	26	0	0
	%C1	0	0	74	63	0
	n	10	7	7	7	7
*hs:fgf8/+* +DM@7.5hpf	%ectopic *six4.1*	100	100	0	0	0

%C1–C5, degree of dorsalization as previously defined [33]; class 1 (C1) is the mildest and class 5 (C5) is the most severe.

**Figure 2:**
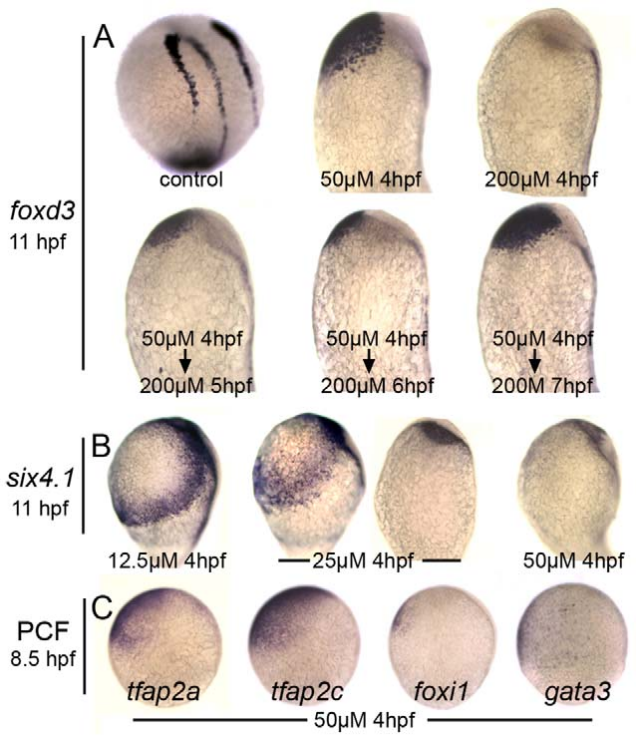
Distinct responses of neural crest and preplacodal ectoderm to graded impairment of Bmp. (A) Lateral views of *foxd3* expression at 11 hpf with anterior up and dorsal to the right. Embryos were treated with indicated concentrations of DM added at 4 hpf. Where indicated the DM concentration was increased to 200 µM (complete Bmp-inhibition) at 5 hpf, 6 hpf or 7 hpf. (B) Lateral views of *six4.1* expression at 11 hpf in embryos treated with indicated concentrations of DM beginning at 4 hpf. Treatment with 25 µM DM yields two discrete responses, one in which *six4.1* remains confined to two bilateral stripes flanking the neural plate and the other in which *six4.1* expression is lost. (C) Lateral views showing expression of preplacodal competence factors *tfap2a*, *tfap2c*, *foxi1* and *gata3* in embryos were treated with 50 µM DM beginning at 4 hpf. Note that *tfap2a/c* remain broadly expressed in ventral ectoderm whereas *foxi1* and *gata3* are nearly eliminated.

Because the role of Bmp in neural crest specification has been well characterized [11]–[13], [15], we tested whether DM could affect this tissue as predicted by these previous studies. Adding 100 or 200 µM DM beginning at 4 hpf totally ablated neural crest formation (Fig. 2A and data not shown). However, adding 50 µM DM at 4 hpf led to ventral expansion of cranial neural crest to fully displace the nonneural ectoderm, similar to the effects of mutations that weaken overall Bmp signaling in zebrafish [12], [13]. These conditions are thought to create a broad plateau of low Bmp signaling appropriate for neural crest specification, providing strong support for the role of Bmp as a morphogen in specifying neural crest. Interestingly, after initially treating embryos with 50 µM DM at 4 hpf, fully blocking Bmp with a super-saturating dose of DM at 5, 6, or 7 hpf does not prevent formation of cranial neural crest, though the domain is somewhat reduced when Bmp is blocked earlier. These data are consistent with the effects of timed misexpression of Chordin [15], showing that Bmp acts very early in cranial neural crest specification and is no longer needed after late blastula/early gastrula stage.

Analysis of preplacodal markers revealed a different pattern of Bmp-dependence. First, preplacodal ectoderm (Fig. 2B) and epidermal ectoderm (not shown) are totally ablated by exposure to 50 µM DM, reflecting loss of all nonneural ectoderm. Accordingly, this treatment eliminated expression of putative preplacodal competence factors *foxi1* and *gata3*, though *tfap2a* and *tfap2*c continue to be expressed (Fig. 2C). The latter two genes are also required in the lateral edges of the neural plate for neural crest development [29], [35]. Second, we found no dose of DM that caused expansion of preplacodal markers throughout the ventral ectoderm. Instead, exposure to 25 µM at 4 hpf yielded two distinct responses; either preplacodal markers were lost entirely or preplacodal ectoderm was shifted ventrally but was still confined to two bilateral stripes bordering the neural plate (Fig. 2B and data not shown). Thus, there does not appear to be a specific level of Bmp that can expand the preplacodal ectoderm at the expense of more ventral (epidermal) ectoderm.

**Figure 3:**
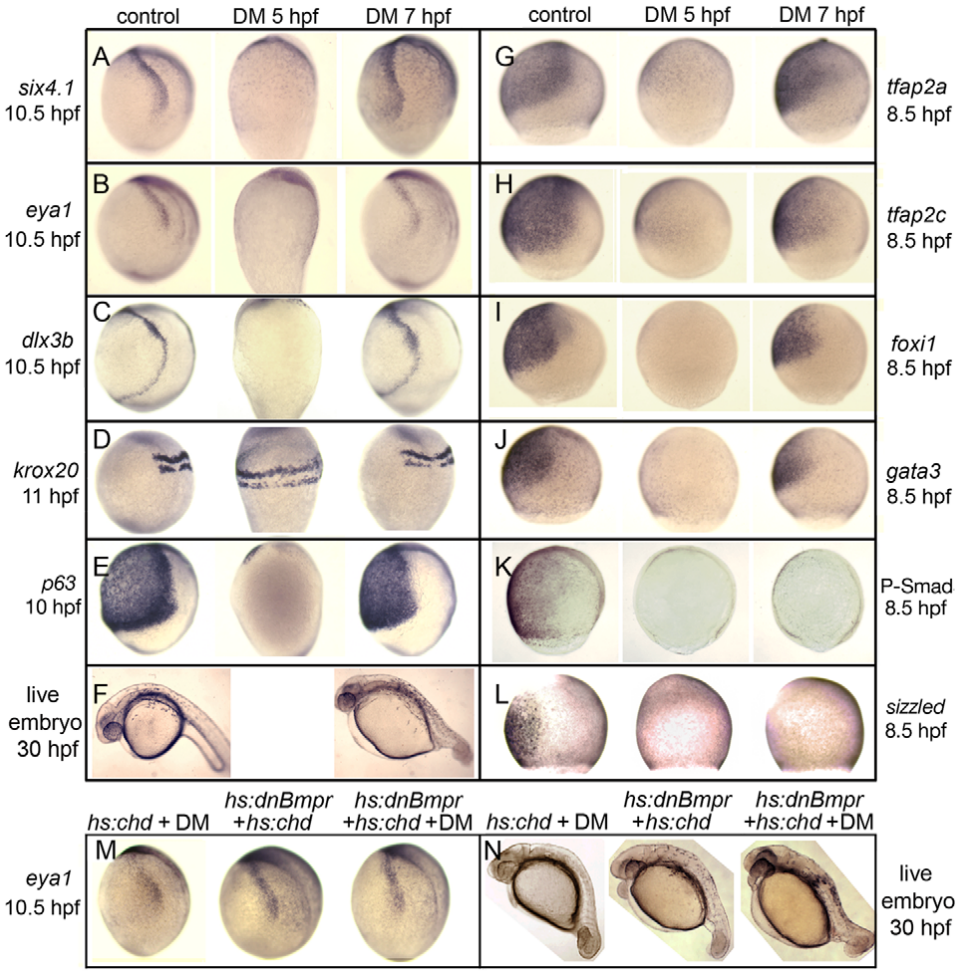
Stage-dependent requirements for Bmp. (A–E, G–L) Analysis of indicated gene expression patterns in control embryos and embryos treated with 100 µM dorsomorphin (DM) at 5 hpf or 7 hpf. Lateral views with dorsal to the right and anterior up. Expression of *six4.1*, *eya1* and *dlx3b* (A–C) in PPE, *krox20* in hindbrain(D) and *p63* in epidermal ectoderm (E). Expression of competence factor genes *tfap2a*, *tfap2c*, *foxi1* and *gata3* (G–J). Reporters of Bmp-signaling, Phospho-Smad1/5/8 antibody staining (K) and *sizzled* in situ hybridization (L). Note the complete loss of Bmp signaling by 100 µM DM-treatment either at 5 hpf or 7 hpf. (F) Lateral views of live embryos at 30 hpf. Embryos treated with DM at 7 hpf show a partially dorsalized C3 phenotype [33]. (M, N) *Tg(hs:chd)* and/or *Tg(hs:dnBmpr)* embryos heat-shocked and treated with 100 µM DM at 7.5 hpf. *eya1* expression (M) and C3 phenotypes (N) are comparable to embryos treated with 100 µM DM alone.

**Figure 4:**
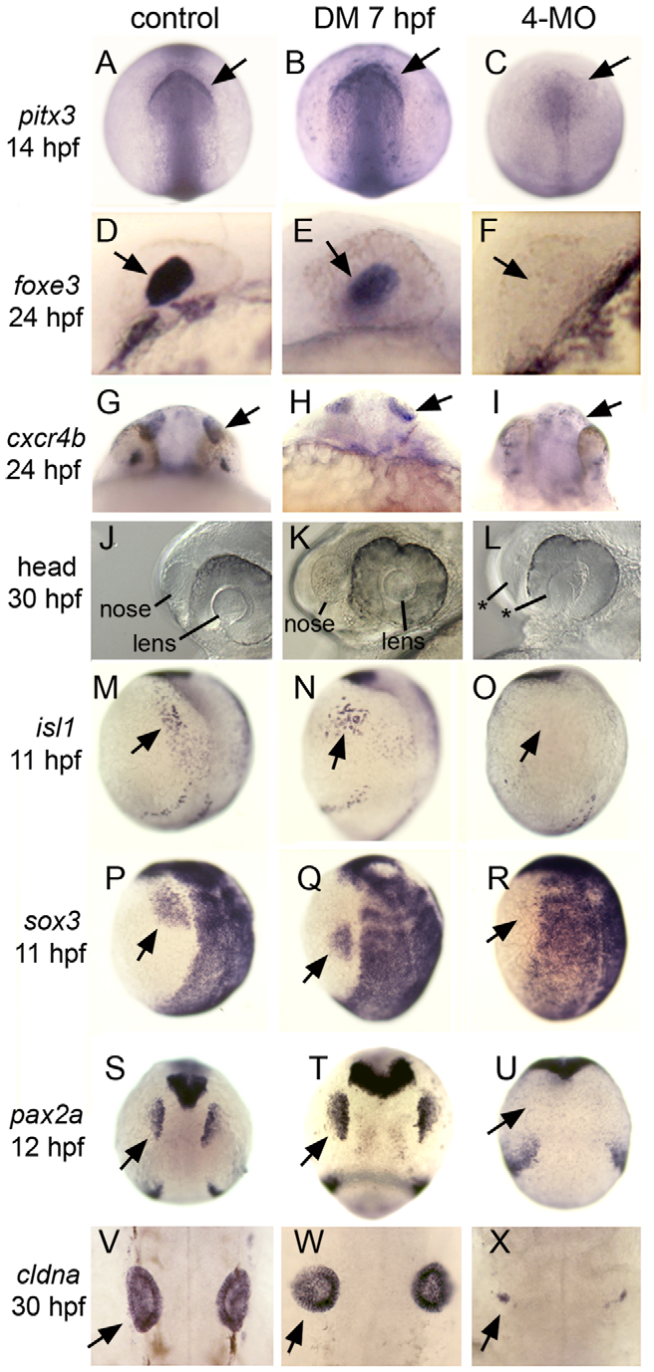
Formation of cranial placodes requires competence factors but not Bmp during gastrulation. Analysis of various cranial placode markers in control embryos, embryos treated with 100 µM DM at 7 hpf, or *foxi1/gata3/tfap2a/c* quadruple morphants (4-MO). Arrows indicate relevant expression domains in placodal tissues. (A–C) Dorsal views (anterior up) of *pitx3* expression in anterior pituitary and lens placode. (D–F) Lateral views (anterior to left) of *foxe3* expression in the lens placode. (G–I) Frontal views of *cxcr4b* expression in olfactory placode. (J–L) Lateral views (anterior to left) showing the lens and nasal pits in live specimens at 30 hpf. Asterisks in (L) depict the absence of morphologically discernable structures. (M–O) Lateral views (anterior up) of *isl1* expression in the trigeminal placode. (P–R) Lateral views (anterior up) of *sox3* expression in the epibranchial placode. (S–U) Dorsal views (anterior up) of *pax2a* expression in the otic placode. (V–X) Dorsal views (anterior up) of *cldna* expression in the otic vesicle. All placodal markers are expressed normally in DM-treated embryos. Expression of *cldna* is severely deficient in quadruple morphants (X, n  =  13/21) or ablated altogether (8/21, not shown). All other placodal markers are ablated in quadruple morphants (n≥10 for each marker).

To characterize the temporal requirements for Bmp, embryos were treated with 100 µM DM at different times during late blastula and early gastrula stages and subsequently analyzed for expression patterns of various ectodermal markers. As expected from the severe dorsalization caused by administering this dose at 5 hpf (Table 1), neural markers were expanded throughout the ectoderm and all nonneural markers were lost, including putative preplacodal competence factors (Fig. 3D, E, G–J). Additionally, definitive preplacodal markers *dlx3b*, *eya1* and *six4.1* were not expressed in these embryos (Fig. 3A–C). In contrast, exposure to 100 µM DM from 7 hpf resulted in only partial dorsalization (Table 1, Fig. 3D, F) and all embryos expressed nonneural markers, albeit in diminished ventral domains (Fig. 3E, G–J). Preplacodal markers *dlx3b*, *eya1* and *six4.1* were expressed on time by 10.5 hpf (Fig. 3A–C). Moreover, all placodal derivatives were produced on time in embryos treated with 100 µM DM from 7 hpf, including the anterior pituitary, olfactory, lens, trigeminal, epibranchial and otic placodes (Fig. 4B, E, H, K, N, Q, T, W) [36]–[46]. Adding 100 µM DM at 6 hpf yielded two classes of embryos, with roughly half being fully dorsalized and the rest resembling the partially dorsalized embryos obtained with 100 µM DM at 7 hpf (Fig. S2, Table 1). Adding 100 µM DM at 5.5 hpf eliminated *eya1* and *six4.1* expression in all embryos, though some embryos still expressed *dlx3b* in bilateral stripes (Fig. S2). These data indicate that embryos make a transition around 5.5–6 hpf after which Bmp is no longer required for preplacodal development. As with treatment during blastula stage, treatment with 100 µM DM during gastrulation eliminated phospho-Smad1/5/8 accumulation and *sizzled* expression, confirming loss of Bmp signaling [15], [34] (Fig. 3K, L). Additionally, the effects of adding 100 µM DM at 7 hpf were identical to the effects of 500 µM DM, the highest dose tested (data not shown), arguing that the block to Bmp signaling was saturated at these doses. Nevertheless, to ensure that Bmp was fully blocked, we combined addition of 100 µM DM at 7 hpf with activation of heat shock-inducible transgenes encoding Chordin and/or dominant-negative Bmp receptor [15], [45] (Fig. 3M, N). The effects on preplacodal specification and morphological development were identical to treatment with 100 µM DM alone. These data show that Bmp is not directly required after the onset of gastrulation for preplacodal specification. The data further show that Bmp signaling is required to induce expression of putative competence factors *foxi1*, *gata3*, *tfap2a* and *tfap2c* during blastula stage, but is not required to maintain them thereafter (Fig. 3G–J).

### Requirement for ventrally expressed competence factors

**Figure 5:**
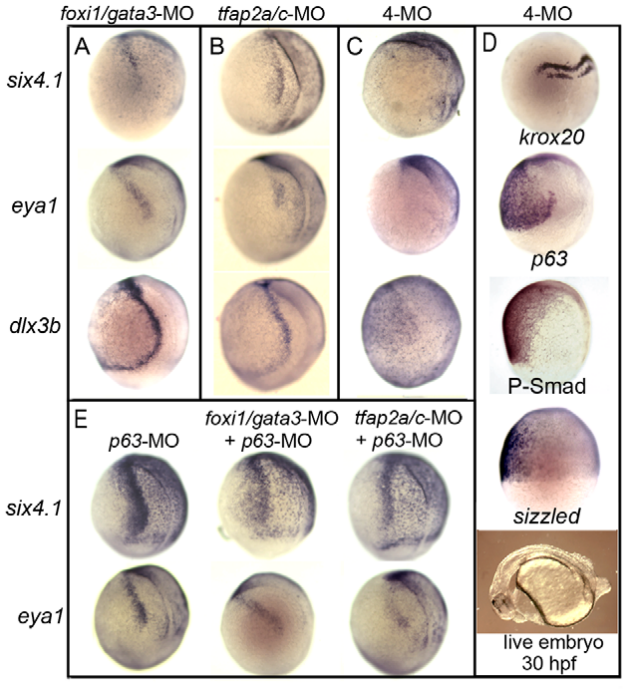
Knockdown of competence factors impairs preplacodal specification. (A–C) Expression of preplacodal markers at 10.5 hpf in (A) *foxi1/gata3* double morphants, (B) *tfap2a/c* double morphants, (C) *foxi1/gata3/tfap2a/c* quadruple morphants (4-MO). Note the complete loss of preplacodal markers in C. (D) Expression of *krox20*, *p63*, P-smad and *sizzled* during gastrulation in *foxi1/gata3/tfap2a/c* quadruple morphants. Morphology of a live quadruple morphant at 30 hpf is also shown. (E) Expression of *six4.1* and *eya1* in *p63* morphants alone or in combination with *tfap2a*/*c*-MO or *foxi1*/*gata3*-MO. All images show lateral views with dorsal to the right and anterior up, except for the live specimen in (D), which shows a lateral view with anterior to the left.

We hypothesized that *foxi1*, *gata3*, *tfap2a* and *tfap2c* encode preplacodal competence factors because they are expressed early throughout the nonneural ectoderm yet are specifically required for later development of various subsets of placodes [24]–[29]. To test the functions of these genes, we injected morpholino oligomers (MOs) to knockdown their functions. Knockdown of any one gene had no discernable effect on preplacodal gene expression (data not shown), though loss of *foxi1* specifically impairs development of the otic and epibranchial placodes [27], [28]. Knockdown of both *foxi1* and *gata3* enhanced the otic placode deficiency (data not shown), and caused a slight reduction in expression levels of *dlx3b*, *eya1* and *six4.1* (Fig. 5A). Knockdown of both *tfap2a* and *tfap2c* caused a stronger reduction in expression levels of preplacodal markers (Fig. 5B). Co-injecting either *gata3*-MO or *foxi1*-MO with *tfap2a/c*-MOs further reduced preplacodal gene expression (data not shown) whereas simultaneous knockdown of *foxi1*, *gata3*, *tfap2a* and *tfap2c* (quadruple morphants) resulted in complete loss of preplacodal gene expression (Fig. 5C). Moreover, development of all cranial placodes (pituitary, olfactory, lens, trigeminal, otic and epibranchial) was severely deficient or totally ablated in all quadruple morphants examined (Fig. 4C, F, I, L, O, R, U, X). Disruption of preplacodal development in quadruple morphants did not reflect general impairment of nonneural ectoderm, as the epidermal marker *p63* [46], [47] was appropriately expressed in the ventral ectoderm (Fig. 5D). Additionally, quadruple morphants did not exhibit elevated cell death, as indicated by relatively normal levels of staining with the vital dye acridine orange [48] (data not shown). These data show that *foxi1*, *gata3*, *tfap2a* and *tfap2c* are specifically required for formation of preplacodal ectoderm and all placodal derivatives, and are partially redundant in this function.

Importantly, quadruple morphants retained a neural-nonneural interface (Fig. 4R and Fig. 5D), the region normally associated with preplacodal specification. Moreover, Bmp signaling also persisted in quadruple morphants as shown by continued ventral accumulation of phospho-Smad1/5/8 and expression of *sizzled* (Fig. 5D). Expression of *fgf3*, *fgf8* and the Fgf-target gene *erm* were also appropriately localized in quadruple morphants (data not shown). Thus, neither Bmp signaling, Fgf signaling, nor neural-nonneural interactions are sufficient for preplacodal specification in this background. These data support the hypothesis that *foxi1*, *gata3*, *tfap2a* and *tfap2c* are required for preplacodal competence or early differentiation.

Although *p63* is normally co-expressed with preplacodal competence factors and is only known to regulate epidermal development [46], [47], we examined whether it is required for preplacodal development. Knockdown of *p63* did not detectably alter preplacodal development, nor did it enhance the deficits in preplacodal gene expression or morphological development seen in *foxi1-gata3* or *tfap2a/c* double morphants (Fig. 5E, and data not shown). This further shows that not all early Bmp-target genes are required for preplacodal development and that the requirement for *foxi1*, *gata3*, *tfap2a* and *tfap2c* is relatively specific.

We also investigated the requirements for *foxi1*, *gata3*, *tfap2a* and *tfap2c* in neural crest formation. Knockdown of both *foxi1* and *gata3* did not alter expression of *foxd3* (data not shown), whereas knockdown of *tfap2a/c* completely eliminated expression of *foxd3* as reported previously [29], [35]. Not surprisingly, *foxd3* expression is also ablated in *foxi1-gata3-tfap2a/c*-quadruple morphants (data not shown). This likely reflects a cell-autonomous requirement for *tfap2a/c* in neural crest specification [29], [35].

### Dorsal misexpression of preplacodal competence factors

**Figure 6:**
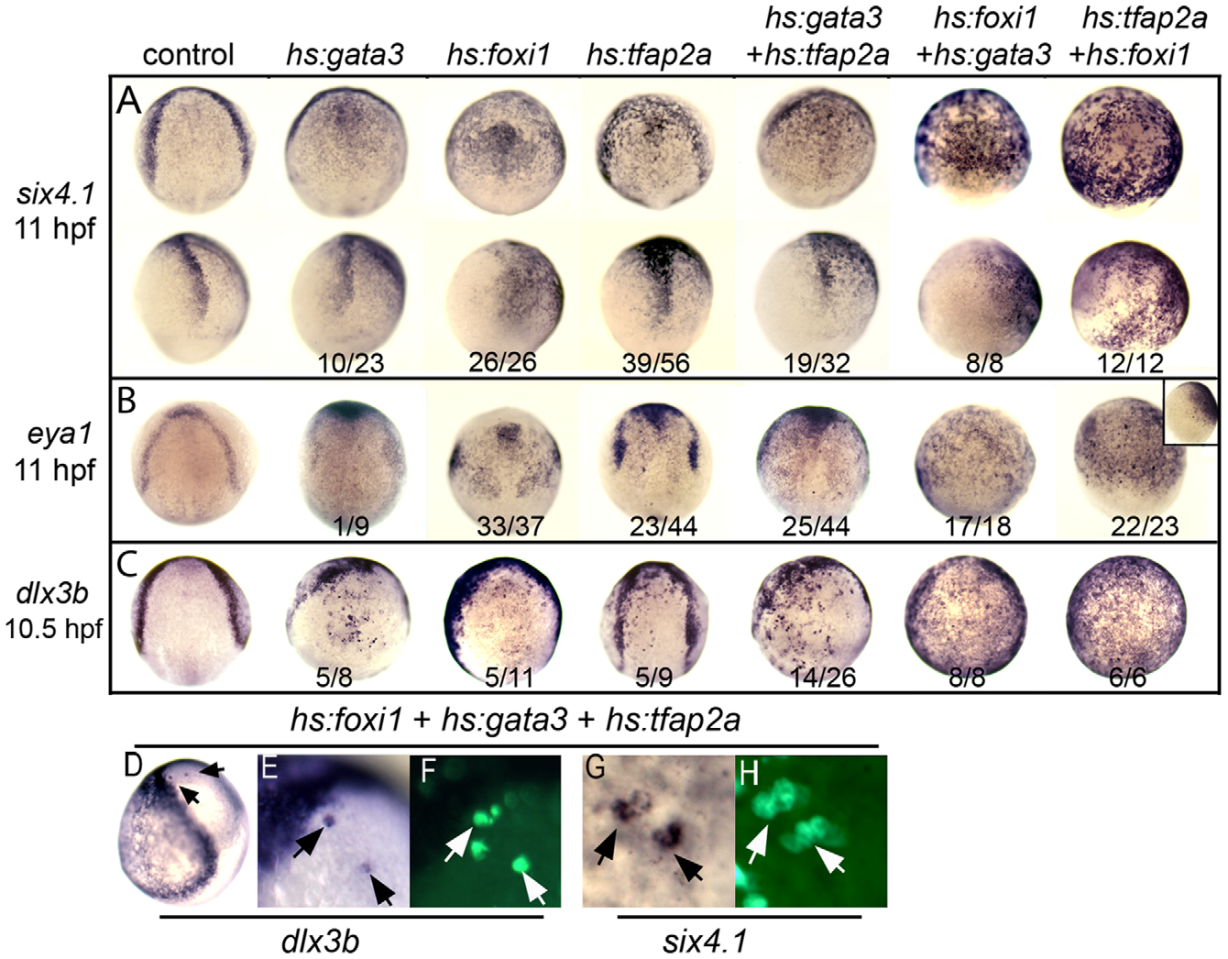
Misexpression of competence factors induces ectopic expression of preplacodal markers. (A–C) Analysis of indicated gene expression patterns in control embryos and embryos carrying *hs:gata3*, *hs:foxi1* and/or *hs:tfap2a* heat-shocked at 30% epiboly (4.5 hpf). Dorsal views with anterior up except bottom row in A, inset in B, which are lateral views with dorsal to the right. Note the ectopic expression of PPE markers, *six4.1* (A), *eya1* (B) and *dlx3b* (C) in neuroectoderm of embryos misexpressing one or more competence factors. (D–H) Dorsolateral views (anterior up) of mosaic embryos showing ectopic expression of *dlx3b* and *six4.1* at 10.5 hpf. Donor cells obtained from *Tg(hs:foxi1)* injected with *hs:gata3* and *hs:tfap2a* plasmid were transplanted into wild type hosts and heat shocked at 4.5 hpf at 39°C. Transplanted cells were identified with Strepavidin-FITC (arrows F, H). Mosaic embryos shows cell autonomous expression of *dlx3b* and *six4.1* in the neural plate (compare E, F and G, H).

To further test the functions of preplacodal competence factors, we generated constructs to misexpress *foxi1*, *gata3* and *tfap2a* under the control of the *hsp70* heat shock promoter [49]. We reasoned that if these genes provide preplacodal competence, then misexpressing them in dorsal ectoderm, where preplacodal inducing factors are normally expressed, should be sufficient to induce ectopic expression of preplacodal genes. We performed transient transfections to introduce *hs:tfap2a* and *hs:gata3* whereas a stable transgenic line was used for *hs:foxi1* (see Materials & Methods). Global heat shock-activation of any one of these genes at 4.5 hpf (late blastula) or 5.5 hpf (early gastrula) resulted in scattered ectopic expression of preplacodal markers within the neural plate by 11 hpf (Fig. 6A–C, and data not shown). In most experiments, over half of embryos showed ectopic expression of preplacodal genes. Co-activation of any two heat shock genes yielded more robust and widespread expression of preplacodal genes in the neural plate, with nearly complete penetrance in most experiments. For reasons that are unclear, misexpression of competence factors at these stages caused widening of the neural plate and narrowing of the ventral Bmp signaling domain (Fig. S3). Nevertheless, Bmp signaling and general DV patterning are still evident following activation of *hs:foxi1*, *hs:gata3* and/or *hs:tfap2a* (Fig. S3). Importantly, we never observed ectopic expression of the epidermal marker *p63* in the neural plate following misexpression of competence factors, indicating that preplacodal competence factors do not induce all nonneural fates in this domain. Co-activation of all three transgenes at 4.5 hpf led to widespread expression of preplacodal genes, but also caused severe axial patterning defects during gastrulation, making results difficult to interpret (data not shown). However, mosaic misexpression of all three competence factors at 4.5 hpf avoided defects in axial patterning yet still led to dorsal expression of *dlx3b* and *six4.1* in a subset of misexpressing cells (Fig. 6D). These data are consistent with the hypothesis that *foxi1*, *gata3* and *tfap2a* are sufficient to render dorsal ectoderm competent to express preplacodal genes in response to dorsally expressed inducing factors.

In addition to their role in preplacodal development, Tfap2a and Tfap2c are required for neural crest [29], [35], whereas Foxi1 and Gata3 are required for preplacodal ectoderm but not neural crest. We asked whether these differing roles in neural crest could also be distinguished in misexpression experiments. Similar to the effects of injecting *tfap2a* mRNA [29], we found that misexpression of *hs:tfap2a*, either alone or in combination with other competence factors, resulted in ectopic *foxd3* expression in the neural plate (Fig. S4). In contrast, activation of *hs:foxi1* and/or *hs:gata3* did not induce ectopic *foxd3* expression (data not shown), but instead reduced expression of *foxd3* in the endogenous neural crest domain (Fig. S4). Importantly, these findings show that formation of ectopic preplacodal tissue is not always associated with neural crest, further arguing that preplacodal competence can be regulated independently from other ectodermal fates.

### Ventral misexpression of preplacodal-inducing factors

**Figure 7:**
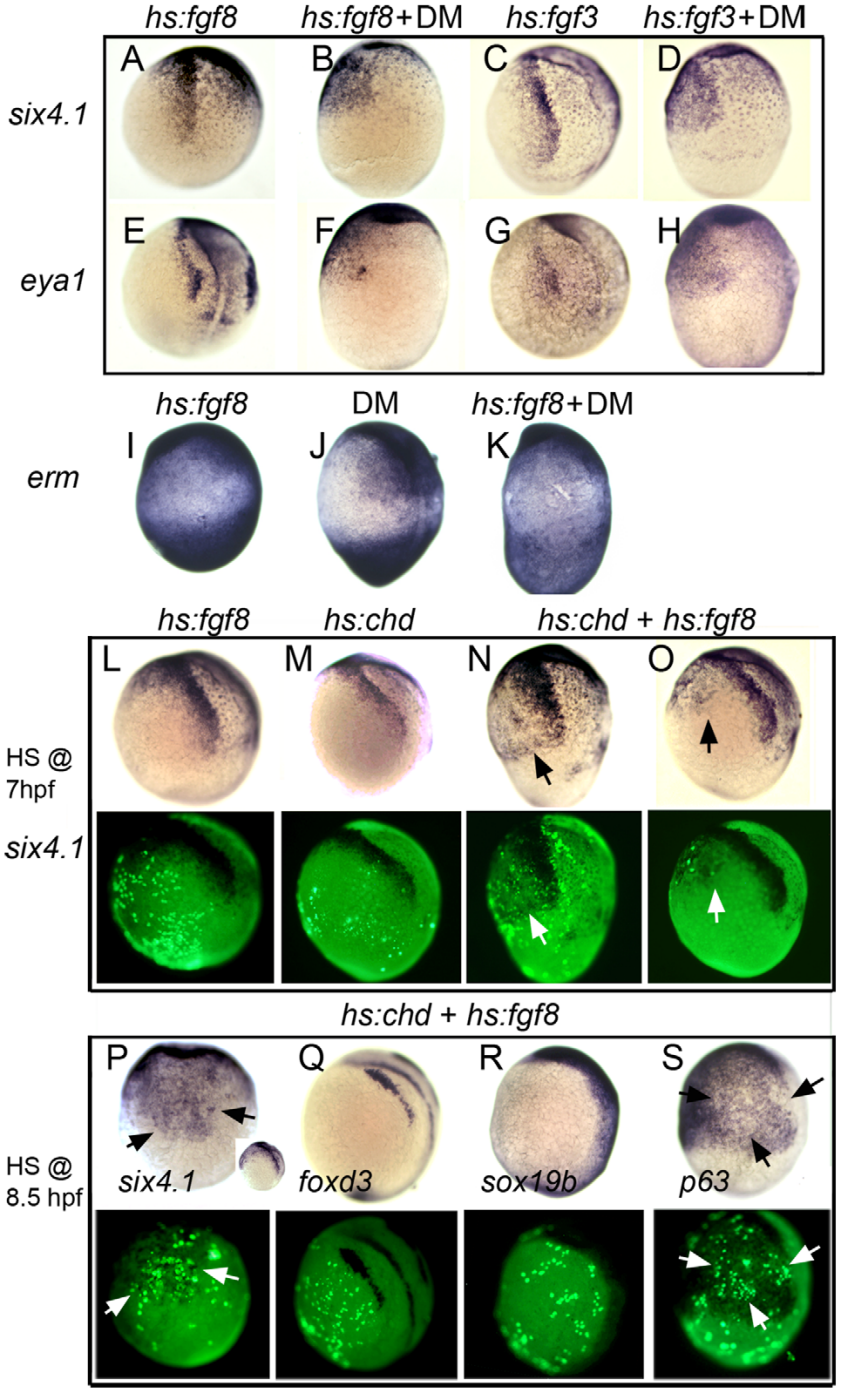
The entire nonneural ectoderm is competent to form preplacodal tissue. (A–H) Expression of preplacodal markers in (A, B, E, F) *Tg(hs:fgf8)* embryos incubated at 35°C from 7.5–10.5 hpf, or (C, D, G, H) *Tg(hs:fgf3)* embryos incubated at 36°C from 7–10.5 hpf. 100 µM DM was added as indicated. (I–K) Expression of *erm* in (I) *Tg(hs:fgf8)* embryo incubated at 35°C without DM, (J) a non-transgenic embryos incubated at 35°C with 100 µM DM, and (K) a *Tg(hs:fgf8)* embryo incubated at 35°C with 100 µM DM. (L–S) Mosaic misexpression of Fgf8 and/or Chordin. (L–O) Brightfield images (top row) and fluorescent images (bottom row) of host embryos with cells transplanted from *Tg(hs:fgf8)* (L), *Tg(hs:chd)* (M) or *Tg(hs:fgf8)*; *Tg(hs:chd)* donor embryos (N, O). Donor embryos were injected with lineage tracer (biotin-dextran) and transplanted at mid-blastula (L, M, N) or early gastrula stage (O) into unlabeled host embryos. Embryos were heat-shocked at 39°C for 30 minutes at 7 hpf and examined for *six4.1* expression at 10.5 hpf. Transplanted transgenic cells were identified by Strepavidin-FITC staining after in situ hybridization. All panels show lateral views of host embryos with anterior up. Mosaic embryos with *Tg(hs:fgf8)*;*Tg(hs:chd)* double transgenic cells showed ectopic *six4.1* expression in surrounding ventral ectoderm (N, O), whereas no ectopic *six4.1* expression was detected following activation of *hs:fgf8* alone (L) or *hs:chd* alone (M). (P–S) Brightfield images (top row) and fluorescent images (bottom row) of host embryos with cells transplanted during early gastrula stage from double heterozygous *Tg(hs:fgf8)*; *Tg(hs:chd)* embryos. Embryos were heat shocked for 30 minutes at 39°C beginning at 8.5 hpf and examined for expression of *six4.1* (preplacodal ectoderm), *foxd3* (neural crest), *sox19b* (neural plate) or *p63*(epidermal ectoderm) at 10.5 hpf. All panels show lateral views except (P) which shows a ventral view (lateral view in inset) and (S) which shows ventro-lateral view. Heat shock activation at 8.5 hpf (P) leads to stronger ectopic expression of *six4.1* than heat shock at 7 hpf (O). No ectopic expression of *foxd3* or *sox19b* is detected (Q, R) whereas *p63* expression appears downregulated in and around transgenic cells (arrows in S).

We next attempted to induce preplacodal development throughout the zone of competence in the nonneural ectoderm by providing appropriate inductive signals normally limited to dorsal tissue. Previous studies have implicated dorsally expressed Bmp-antagonists and Fgfs as preplacodal inducers [16]–[21]. To mimic such signals throughout the nonneural ectoderm, we used heat shock-inducible transgenic lines to misexpress Fgf3 or Fgf8 (*hs:fgf3* and *hs:fgf8*) while blocking Bmp with DM. Using standard heat shock conditions (39°C for 30 minutes) to activate *hs:fgf8* combined with DM treatment at 7.5 hpf fully dorsalized the embryo and was not informative. However, full dorsalization was avoided by prolonged incubation at more moderate temperatures, achieving a weaker level of transgene activation. Incubating *hs:fgf8/+* transgenic embryos at 35°C with 100 µM DM from 7.5–10.5 hpf resulted in expression of *eya1* and *six4.1* throughout the nonneural ectoderm in all embryos (Fig. 7B, F). Diffuse ectopic expression of *erm* confirmed that this heat shock regimen elevated Fgf signaling within nonneural ectoderm (Fig. 7I–K). Similar results were obtained with *hs:fgf3/+* transgenic embryos incubated at 36°C with 100 µM DM from 7–10.5 hpf (Fig. 7D, H). Activation of *hs:fgf3* or *hs:fgf8* alone was not sufficient to activate ectopic preplacodal gene expression (Fig. 7A, C, E, G). These data show that the entire nonneural ectoderm is competent to express preplacodal genes in response to Fgf plus inhibition of Bmp.

We next titrated the dose of DM required for ectopic induction of preplacodal genes. Incubating *hs:fgf8/+* embryos at 35°C with 50 µM DM at 7 hpf led to ventral expression of preplacodal genes, but lower concentrations of DM were not sufficient (Table 1). The finding that 25 µM DM is not sufficient indicates that even very low levels of Bmp signaling can block preplacodal gene activation.

To express inductive signals with greater spatial control, we generated mosaic embryos to locally co-misexpress Fgf8 and Chordin. Donor cells carrying both *hs:fgf8* and *hs:chd* transgenes were transplanted into non-transgenic host embryos at the mid-blastula stage to obtain a random distribution of misexpressing cells. To achieve maximal transgene activation, mosaics were heat-shocked at 39°C for 30 minutes beginning at 7 hpf and then maintained at 33°C until tailbud stage (10 hpf). Of 4 mosaic embryos harboring transgenic donor cells on the ventral side, all showed significant ventral expression of *six4.1* in surrounding host cells (Fig. 7N). In another experiment, transgenic donor cells were transplanted directly to the ventral side at the early gastrula stage (6 hpf). Following heat shock at 7 hpf, all mosaic embryos (n  =  4) showed ectopic *six4.1* expression in surrounding host cells (Fig. 7O). In contrast, no ectopic *six4.1* expression was seen following mosaic misexpression of *hs:fgf8* alone (n  =  13) or *hs:chd* alone (n  =  10) (Fig. 7L, 7M). This confirms that both Fgf and Bmp-antagonists are required to induce expression of preplacodal genes.

Because preplacodal specification has been reported to occur near the end of gastrulation in frog and chick embryos [20], [21], we tested whether activation of *hs:fgf8*; *hs:chd* cells at later stages could also stimulate ectopic preplacodal gene expression. Heat shock activation of ventrally transplanted transgenic cells at 8.5 hpf (yielding peak transgene expression at 9 hpf) led to robust ectopic expression of *six4.1* in surrounding host ectoderm by 11 hpf (Fig. 7P). This suggests that in zebrafish, too, preplacodal specification occurs near the end of gastrulation.

Importantly, activation of *hs:fgf8* and *hs:chd* did not lead to ectopic expression of the general neural plate marker *sox19b* nor the neural crest marker *foxd3* (Fig. 7Q, R). Thus, induction of ectopic *six4.1* expression did not result indirectly from ectopic formation of neural plate. On the other hand, activating transgenic cells at 8.5 hpf caused downregulation of *p63*, suggesting that nearby host cells lose epidermal identity in response to preplacodal specifying signals.

Finally, we reassessed the requirement for Fgf during normal preplacodal specification. Previous studies have reported that expression of preplacodal markers does not require Fgf in zebrafish [50]–[53]. We find that blocking Fgf by adding the pharmacological inhibitor SU5402 at 8.5 hpf did not block expression of preplacodal markers, but levels of expression were reduced (Fig. S5). We speculated that Pdgf, which is also dorsally expressed near the end of gastrulation [54] and activates a similar signal transduction pathway, might provide redundancy with Fgf. We tested this by applying another inhibitor, AG1295, which blocks Pdgf activity in zebrafish [55]. Treatment with AG1295 alone had little effect on preplacodal gene expression, but co-incubation with AG1295 and SU5402 from 8.5 hpf led to further reduction of preplacodal gene expression (Fig. S5). Indeed, expression of *eya1* was almost totally eliminated in the preplacodal domain, though robust expression continues in the cranial mesoderm. These data support the hypothesis that Fgf and Pdgf are partially redundant dorsal factors required for preplacodal specification.

## Discussion

**Figure 8:**
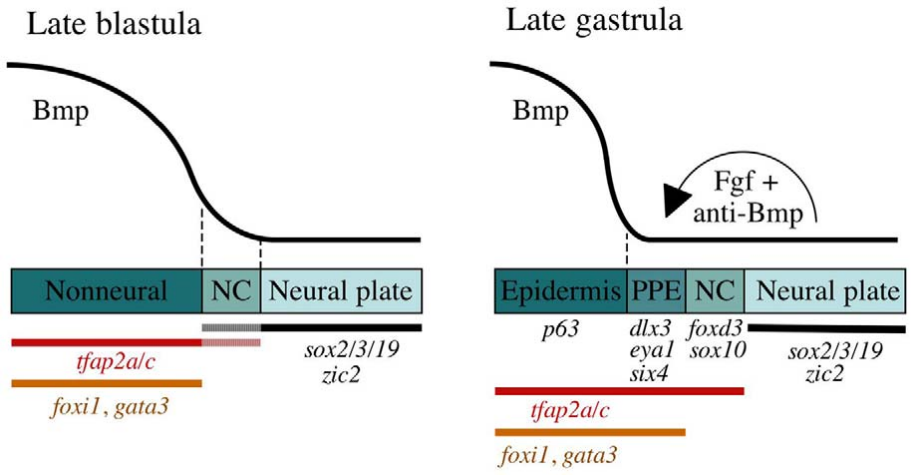
A model for sequential phases of preplacodal development. During late blastula stage, Bmp acts as a morphogen that specifies neural crest (NC) within a narrow but low range of signaling, whereas higher levels of Bmp signaling establish the nonneural ectoderm as a broad zone of uncommitted cells with potential to form epidermal or preplacodal ectoderm (PPE). Within the nonneural ectoderm, changing levels of Bmp do not distinguish preplacodal from epidermal potential, and preplacodal competence factors are uniformly induced throughout this domain. However, expression of *tfap2a/c* overlaps with the lateral edges of the neural plate where, perhaps in combination with neural markers, they cell-autonomously specify NC fate. During late gastrula stage (9–10 hpf), PPE fate is specified in competent cells near the neural-nonneural border by dorsally expressed Bmp antagonists, Fgf and Pdgf. Complete attenuation of Bmp is required for PPE specification. Relevant markers for each ectodermal domain are shown.

We have presented data supporting a relatively simple two-step model of preplacodal development (Fig. 8). First, during late blastula/early gastrula stage Bmp establishes a broad zone of preplacodal competence throughout the nonneural ectoderm. Second, near the end of gastrulation signals from dorsal tissue locally specify preplacodal ectoderm bordering the anterior neural plate. Interestingly, Nguyen et al. proposed a broadly similar two-step model based on analysis of Bmp-pathway mutants in zebrafish [12]. However, at that time neither the molecular basis of preplacodal competence nor the signals required for preplacodal specification were known. Additionally, more recent studies have led to disagreement as to whether Bmp is required at a specific low level or must be blocked entirely for preplacodal specification [18]–[21]. Our model resolves the role of Bmp, confirms that Fgf plus Bmp-antagonists are sufficient for preplacodal specification, shows for the first time that Fgf and Pdgf cooperate as redundant preplacodal inducing factors, and highlights the importance of Foxi1, Gata3, Tfap2a and Tfap2c as preplacodal competence factors. We also readdress mechanisms of neural crest specification, which show a number of crucial differences from preplacodal ectoderm.

### Distinct roles for Bmp in specification of neural crest and preplacodal ectoderm

Using DM to finely control Bmp signaling, we show that Bmp regulates neural crest and preplacodal ectoderm by markedly different mechanisms. In agreement with earlier genetic studies in zebrafish [12], [13], [15], our data indicate that neural crest is specified by a discrete low level of Bmp signaling as predicted by the classical morphogen model (Fig. 1A). Adding DM at 4 hpf at a dose sufficient to fully block Bmp signaling ablates neural crest formation, whereas a slightly lower dose causes a dramatic ventrolateral expansion of neural crest to fully displace nonneural ectoderm (Fig. 2A). Fully blocking Bmp after the onset of gastrulation does not block neural crest, in agreement with studies involving timed misexpression of Chordin [15]. These data suggest that cranial neural crest is already specified by early gastrula stage, after which it no longer requires Bmp. In chick, too, neural crest is specified by early gastrula stage [22].

Preplacodal ectoderm, marked by expression of *dlx3b*, *eya1* and *six4.1*, develops in two distinct phases with distinct signaling requirements, neither of which resemble the pattern shown by neural crest. Preplacodal ectoderm requires a robust Bmp signal during late blastula/early gastrula, but unlike neural crest, there does not appear to be a specific range of Bmp signaling that uniquely specifies preplacodal fate. We found no dose of DM that could expand the preplacodal ectoderm in a manner similar to neural crest. Instead, increasing the concentration of DM (lowering Bmp signaling) either shifted discrete bilateral stripes of preplacodal ectoderm to a more ventral position or eliminated them altogether, depending on the degree of neural plate expansion. Indeed, treatment with a single dose (25µM DM beginning at 4 hpf) yielded both classes of embryo, with nothing in between. Thus, DM cannot expand preplacodal ectoderm at the expense of epidermal ectoderm, indicating that changing Bmp levels do not distinguish between these fates.

The requirement for Bmp changes during the second phase of preplacodal development beginning soon after the onset of gastrulation. Adding a full blocking dose of DM at 7 hpf does not block preplacodal specification, even if transgenic Chordin and dominant-negative Bmp receptor are also activated during this period. Thus, Bmp is not required during gastrulation for preplacodal specification. By extension, the requirement of preplacodal ectoderm for locally secreted Bmp-antagonists [16]–[21] cannot reflect a requirement for a specific low threshold of Bmp; instead Bmp-antagonists are presumably needed to fully attenuate Bmp. This conclusion is further supported by our experiments showing that a full blocking dose of DM is required to induce ectopic preplacodal markers throughout the ventral ectoderm (Fig. 7, Table 1, and see below).

### Other essential signals

We have found that Fgf combined with Bmp attenuation is sufficient to induce preplacodal markers in ventral ectoderm, as has been shown in chick and frog [20], [21], suggesting that this mechanism is broadly conserved. Thus, using heat shock-inducible transgenes, we show that misexpression of Fgf combined with DM treatment is sufficient to induce ectopic preplacodal markers anywhere within the nonneural ectoderm. This supports two important conclusions. First, it demonstrates that the entire nonneural ectoderm is competent to form preplacodal ectoderm, even at the ventral midline far from the neural plate. This is consistent with the expression domains of preplacodal competence factors (see below). Second, although Fgf and Bmp-antagonists likely constitute a small subset of signals associated with the neural-nonneural border, no other signals are needed to trigger preplacodal development. Fgf and Bmp-attenuation induces ectopic expression of preplacodal markers in chick and *Xenopus* [20], [21], though this combination of signals also induces expression of general neural plate markers in those species. By contrast, our experimental conditions do not induce formation of ectopic neural plate or neural crest, tissues that could themselves have induced ectopic preplacodal markers [20], [21], [23]. Thus induction of ectopic preplacodal ectoderm appears to be a direct and specific response to Fgf combined with Bmp attenuation, at least in zebrafish.

In addition to being able to induce ectopic preplacodal markers, we have found that Fgf is required in zebrafish for normal preplacodal development, and furthermore that Pdgf acts partially redundantly in this process. Fgf and Pdgf have been shown to regulate distinct aspects of gastrulation, with Fgf promoting dorsal fate specification and Pdgf promoting convergence towards the dorsal midline [55], [56]. Although Fgf is not absolutely required for expression of general preplacodal markers [50]–[53], we find that treating embryos with the Fgf inhibitor SU5402 during the latter half of gastrulation reduces the level of expression of preplacodal markers. Treating embryos with the Pdgf inhibitor AG1295 alone has no effect on preplacodal specification, but blocking both Fgf and Pdgf further reduces preplacodal gene expression, nearly eliminating *eya1* expression. Homologs of Fgf and Pdgf are preferentially expressed in dorsal tissues near the end of gastrulation [54], [56], [57] and likely activate the same signal transduction pathways required for preplacodal specification. It is not known whether Pdgf regulates preplacodal development in other species, but Pdgf and Fgf are specifically required for induction of the trigeminal placode in chick [58].

In this study we have not addressed the role of Wnt inhibitors, which are also required for preplacodal development [18], [21]. Numerous Wnt inhibitors are abundantly expressed in the head and are vital for cranial development in general, including preplacodal ectoderm. Otherwise, preplacodal fate is restricted from the trunk and tail by posteriorizing Wnt signals [59], [60].

### The role of competence factors

We show that Tfap2a, Tfap2c, Foxi1 and Gata3 act as partially redundant competence factors required specifically for preplacodal development. These genes are expressed uniformly within the nonneural ectoderm beginning in late blastula stage. Knockdown of individual competence factors can impair development of discrete subsets of cranial placodes but formation of preplacodal ectoderm is not detectably altered [24]–[29]. In contrast, knockdown all four competence factors specifically blocks formation of preplacodal ectoderm and all placodal derivatives (Fig. 4, Fig. 5). Importantly, formation of a ventral Bmp gradient and the neural-nonneural interface still occurs. Formation of this region reflects a signaling environment that normally promotes preplacodal development yet, without the four competence factors, cells in the nonneural ectoderm cannot respond to such signals. Conversely, misexpression of one or more competence factors in the neural plate, where preplacodal inducing signals are expressed, leads to ectopic expression of preplacodal markers (Fig. 6). Although global misexpression of competence factors causes various developmental defects, localized mosaic misexpression avoids global perturbation yet still results in cell-autonomous expression of preplacodal markers in the neural plate. Thus, these genes are necessary and sufficient to render cells competent to form preplacodal ectoderm, while additional dorsal signals are required for overt specification of preplacodal fate.

Though *tfap2a/c*, *foxi1* and *gata3* are required for preplacodal ectoderm, they are neither necessary nor sufficient for epidermal fate: Expression of the epidermal marker *p63* remains appropriately localized following either knockdown or misexpression of preplacodal competence factors (Fig. 5, Fig. 6). Conversely, knockdown of *p63* does not detectably impair preplacodal development nor enhance the effects of knocking down subsets of preplacodal competence factors (Fig. 5). The simplest interpretation is that Bmp initially co-induces epidermal and preplacodal potential throughout the nonneural ectoderm, with fate specification occurring later according to differences in local signaling.

Differential regulation of preplacodal competence factors by Bmp explains the differing Bmp-requirements of preplacodal ectoderm vs. neural crest. *tfap2a*, *tfap2c*, *foxi1* and *gata3* all require Bmp for ventral expression during blastula stage. Because these genes are expressed uniformly throughout the nonneural ectoderm, it is now clear why no dose of DM is capable of expanding preplacodal ectoderm at the expense of epidermal ectoderm, though both fates can be eliminated together at sufficiently high concentrations. However, *tfap2a* and *tfap2c* are expressed in a broader domain that includes the lateral edges of the neural plate where they are required for neural crest specification [29], [35]. The broader domain of expression suggests that *tfap2a* and *tfap2c* can be induced by a lower level of Bmp than *foxi1* and *gata3*. Indeed, we identified a dose of DM that permits continued broad expression of *tfap2a/c* but eliminates expression of *foxi1* and *gata3* (Fig. 2). Thus the greater sensitivity of *tfap2a/c* to Bmp explains the ability of a low threshold of Bmp to expand neural crest at the expense of nonneural ectoderm. After the onset of gastrulation, expression of all four genes becomes independent of Bmp. This is an important regulatory feature because it allows maintenance of preplacodal competence as Bmp signaling is attenuated along the neural-nonneural border during preplacodal specification. Likewise, stability of *tfap2a/c* in the neural plate safeguards neural crest fate after Bmp signaling abates.

It is still unclear how *tfap2a/c* can alternately promote either neural crest or preplacodal development. We speculate that the overlap of *tfap2a/c* with early markers of neural plate such as *sox2/3/19* favors neural crest, whereas overlap with *foxi1* and *gata3* in the nonneural ectoderm favors preplacodal development (Fig. 8). However, misexpression of *tfap2a* in the neural plate can induce both neural crest and preplacodal markers, albeit in non-overlapping clusters of cells (Fig. S4). It is possible that the level of *tfap2a* and *tfpa2c* also influences its developmental function. Both genes show diminishing expression near the edges of the neural plate, which might facilitate their neural crest functions. Similarly, cell-to-cell variation in the level of *hs:tfap2a* transgene expression might explain the ability to activate ectopic preplacodal and neural crest markers in dorsal ectoderm.

The long lag between expression of competence factors and expression of preplacodal markers remains unexplained. That is, why are preplacodal competence factors expressed prior to gastrulation yet preplacodal markers are not induced until the end of gastrulation? We cannot accelerate expression of preplacodal markers by changing the time of activation of *hs:fgf8* and *hs:chd*. Regardless of whether we activated these transgenes at 7 hpf or 8.5 hpf, we only detected ectopic expression of preplacodal markers at 10.5–11 hpf, the same time these genes are induced within the endogenous preplacodal domain. It is possible that competence factors require sufficient time to “condition” ectoderm, for example through chromatin remodeling [61], or by activating other essential co-factors. These are important issues that require further investigation.

## Materials and Methods

### Standard development, staging, and pharmacological inhibitor treatment

Embryos were developed under standard conditions at 28.5°C except where noted and staged according to standard protocols [62]. To block Bmp, dorsomorphin (DM) (Calbiochem, 171260) was added to the fish water from a 10mM stock in DMSO. Embryos were treated without removing their chorions. Treatment was carried out in 24-well plates, with 40 embryos in 0.5 ml of solution per well. Relevant controls were incubated in fish water containing an equal concentration of DMSO to that of treated embryos. DM solutions should be exposed to as little light as possible as the drug is photo-unstable. Stock solution of DM may be stored in small aliquots at −80°C for several months, but storage at warmer temperatures and repeated freeze-thaw significantly reduces activity. To Block Fgf, SU5402 (Calbiochem) was diluted from a 10 mM stock in DMSO. To block Pdgf, AG1295 (Calbiochem) was diluted from a 20mM stock in DMSO.

### *In situ* hybridization and immunostaining

Fixation and *in situ* hybridization were performed as previously described [48], [57]. Immunostaining for phosphorylated Smads was carried out as described [15] with minor modifications. The primary antibody was used at a concentration 1∶150 (anti-pSmad1/5/8 antibody; Cell Signaling Technology). Secondary antibody was HRP-conjugated anti-rabbit IgG at 1∶200 (Santa Cruz Biotechnology).

### Morpholino injection

For gene knockdown experiments, embryos were injected with 5ng per morpholino as indicated. Morpholino sequences for *foxi1*, *tfap2a*, *tfap2c* and *p63* have been previously published [27], [29], [63]. To knockdown *gata3*, either of two morpholinos was used: For blocking translation, *gata3*-MO1 TCCGGACTTACTTCCATCGTTTATT; for blocking mRNA splicing at the exon1-intron1 junction, *gata3*-MO2 AGAACTGGTTTACTTACTGTGAGGT. Neither *gata3*-MO1 nor *gata3*-MO2 produced discernable phenotypes on their own, but both showed identical interactions with morpholinos for other competence factors. The ability of *gata3*-MO2 to diminish production of mature *gata3* mRNA was confirmed with RT-PCR (Fig. S6). The MO-generated phenotypes described in this study were 100% penetrant, except where noted in the text. At least 10 specimens were examined or each experimental time point, unless stated otherwise.

### Gene misexpression

Full length cDNAs of *foxi1*, *gata3*, *tfap2a*, *fgf3* and *fgf8* were ligated to *hsp70* heat shock promoter [49] with flanking *I-SceI* meganuclease sites [64], [65]. Recombinant plasmid (10–40 pg/nl) was coinjected with *I-SceI* meganuclease (NEB, 0.5 U/µl) into 1-cell stage embryos. For transient ectopic expression, injected embryos were heat-shocked in a recirculating water bath. Stable transgenic lines *Tg(hsp70:fgf8a)^x17^*, *Tg(hsp70:fgf3)^x18^* and *Tg(hsp70:foxi1)^x19^* were generated by raising injected embryos to adult and screening by PCR for germline transmission. Heterozygous transgene-carriers were easily distinguished based on the phenotype following heat shock at 30% epiboly: Activation of *Tg(hsp70:fgf8a)^x17^* or *Tg(hsp70:fgf3)^x18^* caused dorsalization of the embryo, whereas activation of *Tg(hsp70:foxi1)^x19^* caused anterior truncations with defects in forebrain and eyes (Fig. S3H). The *Tg(hsp70l:dnBmpr-GFP)* transgenic line [45] was provided by ZIRC. *Tg(hsp70:chordin)* [15] was generously provided by Mary Mullins.

In most experiments, transgenic embryos were heterozygous for the transgenes in question, with the exception that homozygous *Tg*(*hsp70:chordin*)/Tg(hsp70*:chordin*) embryos were used to misexpress *chd*. To misexpress *foxi1*, *tfap2a* and *gata3*, embryos were heat shocked at 39°C for 30 min at various times as indicated in the text. *Tg(hsp70l:dnBmpr-GFP)* and *Tg(hsp70:chordin)* embryos were heat shocked at 39°C for 30 min at 7.5 hpf; *Tg(hsp70:fgf8a)*and *Tg(hsp70:fgf3)* embryos at 35°C for 3 hr from 7.5 hpf. After heat shock, the plate containing the embryos was transferred into a 28.5°C incubator until fixation or observation.

### Cell transplantation

Donor embryos were injected with lineage tracer (mix of lysine fixable rhodamine dextran, 10000 MW, and 5% biotin dextran, 10000 MW, in the ratio of 1∶9 in 0.2 M KCl) at the one-cell stage. Cells were transplanted either from blastula stage donors into blastula stage hosts or from blastula stage donors into gastrula stage (∼6 hpf) hosts. Mosaic embryos were then heat-shocked at 39°C for 30 min at 7 hpf and subsequently maintained at 33°C until fixed. Transplanted cells were identified in the hosts by streptavidin-FITC antibody staining.

### Cell death assays

Embryos were dechorinated and incubated for 1 hour on agarose-coated plates containing fish water with acridine orange (AO) (1µg/ml), as modified from [48]. The embryos were then briefly washed and immediately examined under a fluorescence microscope.

## Supporting Information

Figure S1 Dorsomorphin acts quickly to block Bmp signaling. Embryos were treated with either 1% DMSO (controls) or 100 µM DM beginning at 5 hpf. (A) Phospho-Smad staining in a control after 1 hour, or in DM-treated embryos after 15 minutes or 1 hour. (B) Expression of *sizzled* in a control embryo after 1 hour, or in DM-treated embryos after 30 minutes or 1 hour. All images show animal pole views with dorsal to the right.
(0.58 MB TIF)

Figure S2 Additional data showing the effects of DM on preplacodal development and cell survival. (A, B) Expression of preplacodal markers at 10.5 hpf following addition of 100 µM DM at 5.5 hpf or 6 hpf. Treatment at 5.5 hpf eliminated expression of *eya1* and *six4.1*, whereas *dlx3b* was either lost or expressed in bilateral stripes (the specimens processed for *dlx3b* expression were from the same experiment). Treatment at 6 hpf yielded two classes of embryos, with some showing loss of preplacodal markers and others showing bilateral stripes of preplacodal markers (the two specimens processed for *six4.1* expression were from the same experiment). (C) Dorsal views of embryos stained with acridine orange (AO) at 11 hpf following addition of DMSO (control) or 200 µM DM at 7 hpf. AO staining in is comparable in controls and DM-treated embryos. At least 20 specimens were examined for each marker and time point.
(0.68 MB TIF)

Figure S3 Assessment of general embryonic pattering following global misexpression of competence factors. Plasmid vectors for *hs:tfap2a* or *hs:gata3* were injected into wild-type embryos or *Tg(hs;foxi1)* transgenic embryos, as indicated across the top of the Figure. Embryos were heat shocked at 4.5 hpf, including the non-transgenic controls. A–F, expression of various markers at the indicated times: (A–C) neurectodermal markers *sox19b*, *krox20 and fgf3* [56], [57], (D) Fgf-target gene *erm*, (E) epidermal marker *p63* [46], [47] and (F) Bmp target gene *sizzled* [34]. Misexpression of competence factors does not block Bmp or Fgf signaling nor general features of axial patterning, though embryos appear partially dorsalized. (G) AO staining in the respective transgenic carriers. *hs:gata3* showed reduced cell death while other transgenes alone or in combination resulted in slightly increased cell death compared to control embryos. (H) Lateral views of live embryos at 28 hpf. A–C, E, and G show dorsal views of embryos, D shows dorsolateral views, and F and insets in E show lateral views.
(3.06 MB TIF)

Figure S4 Effect of misexpression of competence factors on neural crest development. (A–D) Expression of *foxd3* at 11 hpf in a control embryo (A), or following activation of *hs:tfap2a* (B), *hs:foxi1* (C) or *hs:gata3* (D) at 4.5 hpf. (E, F) Expression of *six4.1* (blue) and *foxd3* (red, fluorescence) in a control embryo (E) or following activation of *hs:tfap2a* and *hs:foxi1* at 4.5 hpf (F). Scale bar  =  50 µm.
(0.42 MB TIF)

Figure S5 Blocking Fgf and Pdgf signaling leads to downregulation of preplacodal markers. (Upper two rows) Dorsal views showing expression of *eya1* and *dlx3b* at 1l hpf in wild-type embryos that were treated beginning at 8.5 hpf with 15µM AG1295, 25µM SU5402, or both. AG1295 did not cause any significant changes in the expression. SU5402 reduced expression of both genes. Addition of both inhibitors caused loss of *eya1* within the preplacodal domain and significant downregulation of *dlx3b*. (Lower row) Images of live embryos at 24 hpf. Treatment with SU5402 or both SU5402 and AG1295 severely perturbed caudal development and blocked formation of the otic vesicle.
(0.96 MB TIF)

Figure S6 Effects of *p63*-MO and *gata3*-MO2 on accumulation of mature mRNA. (A) *p63*-MO leads to an aberrantly spliced transcript. Control embryos or embryos injected with *p63* splice blocker were lysed at 11 hpf to collect mRNA. Primers for *p63* and a constitutive control, *ornithine decarboxylase* (*odc*) were added to lysates to synthesize cDNA, which was then amplified for 30 cycles. *p63*-MO caused loss of wild-type transcript and accumulation of an aberrant splice product of higher molecular weight. (B) *gata3*-MO2 causes loss of *gata3* transcript. Control embryos or embryos injected with *gata3*-MO2 (splice-blocker) were lysed at 12 hpf to collect mRNA. Primers for *gata3* and *odc* were added to lysates to synthesize cDNA, which was then amplified for 30 cycles. Primers for gata3 flanked the splice junction between exons 1 and 2. Primer sequences: *gata3*: GTGTTGTGTGTATCGGTGAGTG, GAGGAGGAAGAAGCTGGAGG; *odc*: GGATGTCCTGAAGCACCT, CCCACTGACTGCACGAT; *p63*: Primers were the same as those used previously [63].
(0.20 MB TIF)
