# Supplementary figures and images for: Identification of Early Requirements for Preplacodal Ectoderm and Sensory Organ Development
Source: PLoS Genet. 2010 Sep 23;6(9):e1001133. doi: 10.1371/journal.pgen.1001133 (PMC2944784; doi:10.1371/journal.pgen.1001133)

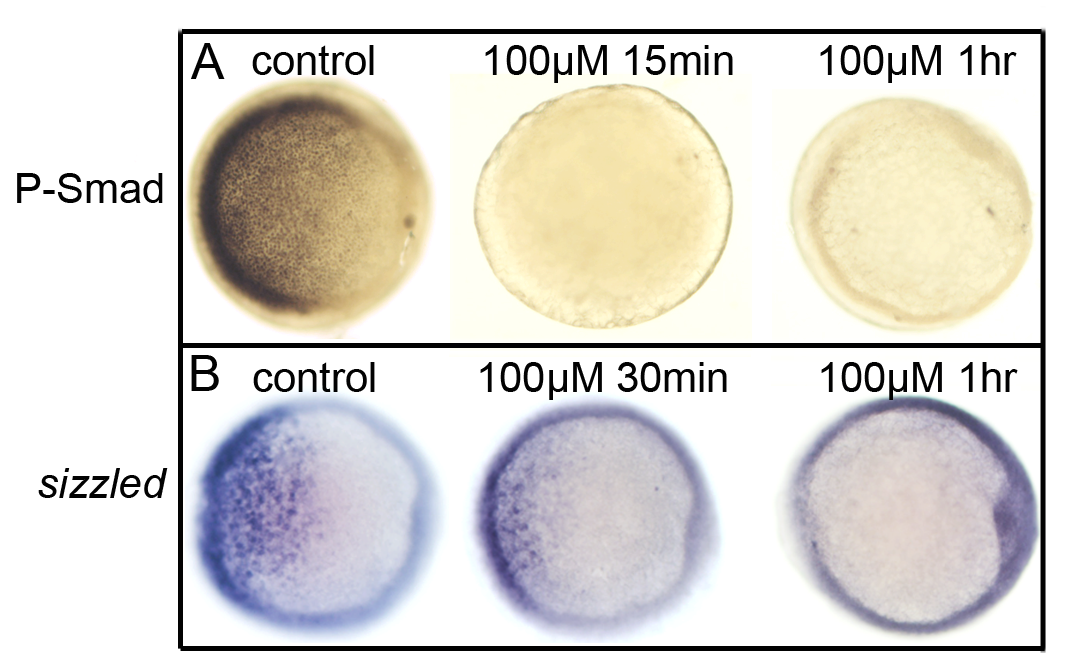

Supplement: Figure S1 — Dorsomorphin acts quickly to block Bmp signaling. Embryos were treated with either 1% DMSO (controls) or 100 µM DM beginning at 5 hpf. (A) Phospho-Smad staining in a control after 1 hour, or in DM-treated embryos after 15 minutes or 1 hour. (B) Expression of sizzled in a control embryo after 1 hour, or in DM-treated embryos after 30 minutes or 1 hour. All images show animal pole views with dorsal to the right. (0.58 MB TIF) [file pgen.1001133.s001.tif]

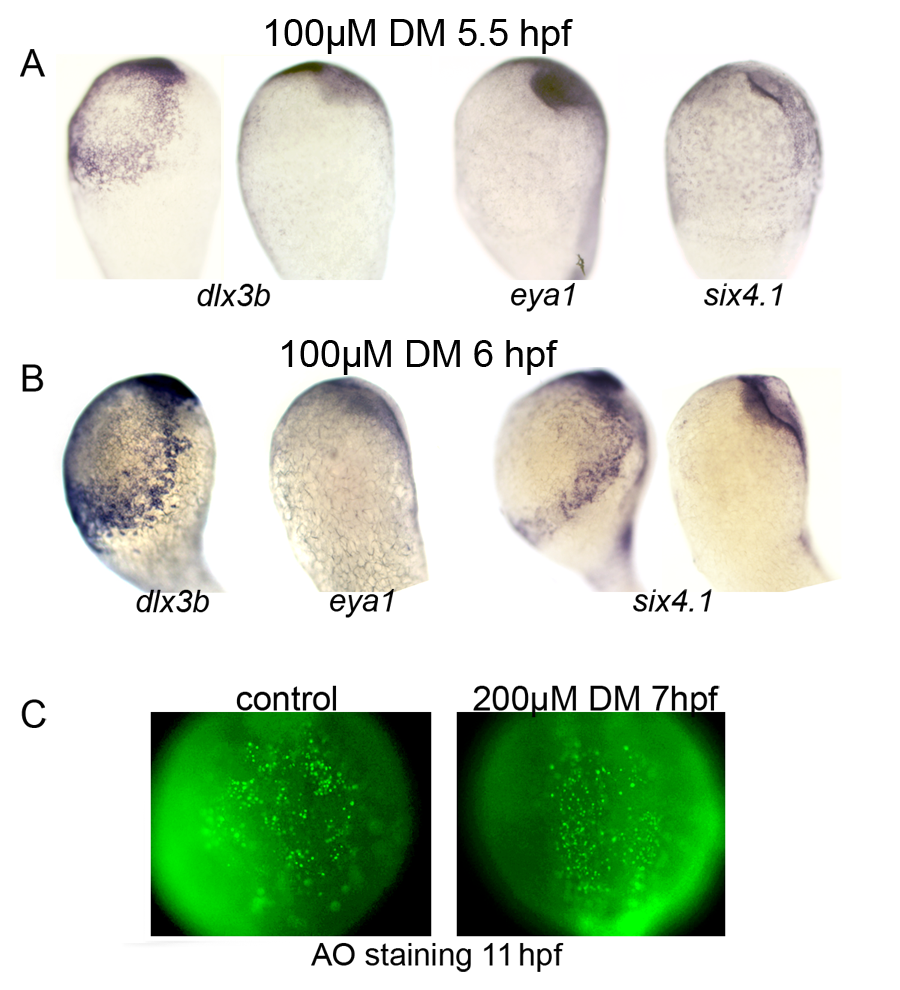

Supplement: Figure S2 — Additional data showing the effects of DM on preplacodal development and cell survival. (A, B) Expression of preplacodal markers at 10.5 hpf following addition of 100 µM DM at 5.5 hpf or 6 hpf. Treatment at 5.5 hpf eliminated expression of eya1 and six4.1, whereas dlx3b was either lost or expressed in bilateral stripes (the specimens processed for dlx3b expression were from the same experiment). Treatment at 6 hpf yielded two classes of embryos, with some showing loss of preplacodal markers and others showing bilateral stripes of preplacodal markers (the two specimens processed for six4.1 expression were from the same experiment). (C) Dorsal views of embryos stained with acridine orange (AO) at 11 hpf following addition of DMSO (control) or 200 µM DM at 7 hpf. AO staining in is comparable in controls and DM-treated embryos. At least 20 specimens were examined for each marker and time point. (0.68 MB TIF) [file pgen.1001133.s002.tif]

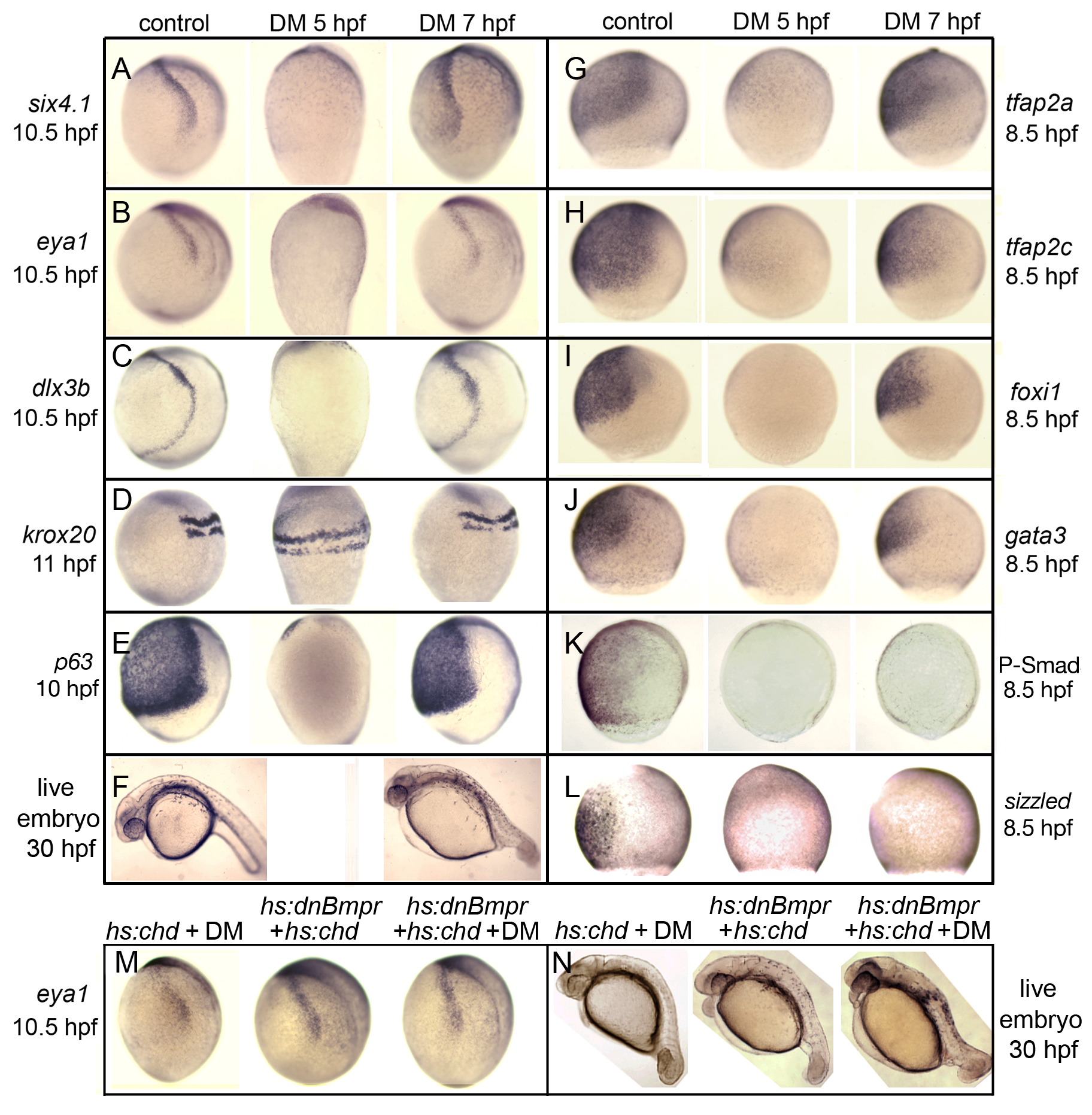

Supplement: Figure S3 — Assessment of general embryonic pattering following global misexpression of competence factors. Plasmid vectors for hs:tfap2a or hs:gata3 were injected into wild-type embryos or Tg(hs;foxi1) transgenic embryos, as indicated across the top of the Figure. Embryos were heat shocked at 4.5 hpf, including the non-transgenic controls. A–F, expression of various markers at the indicated times: (A–C) neurectodermal markers sox19b, krox20 and fgf3 [56], [57], (D) Fgf-target gene erm, (E) epidermal marker p63 [46], [47] and (F) Bmp target gene sizzled [34]. Misexpression of competence factors does not block Bmp or Fgf signaling nor general features of axial patterning, though embryos appear partially dorsalized. (G) AO staining in the respective transgenic carriers. hs:gata3 showed reduced cell death while other transgenes alone or in combination resulted in slightly increased cell death compared to control embryos. (H) Lateral views of live embryos at 28 hpf. A–C, E, and G show dorsal views of embryos, D shows dorsolateral views, and F and insets in E show lateral views. (3.06 MB TIF) [file pgen.1001133.s003.tif]

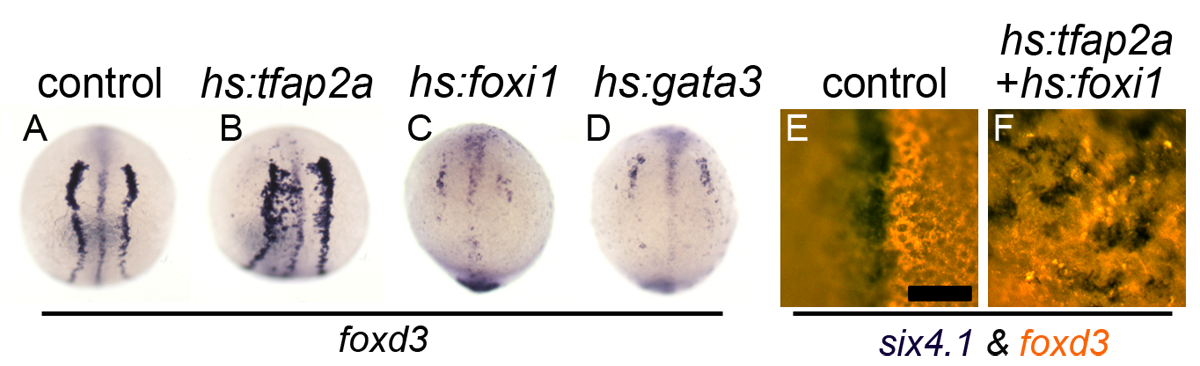

Supplement: Figure S4 — Effect of misexpression of competence factors on neural crest development. (A–D) Expression of foxd3 at 11 hpf in a control embryo (A), or following activation of hs:tfap2a (B), hs:foxi1 (C) or hs:gata3 (D) at 4.5 hpf. (E, F) Expression of six4.1 (blue) and foxd3 (red, fluorescence) in a control embryo (E) or following activation of hs:tfap2a and hs:foxi1 at 4.5 hpf (F). Scale bar = 50 µm. (0.42 MB TIF) [file pgen.1001133.s004.tif]

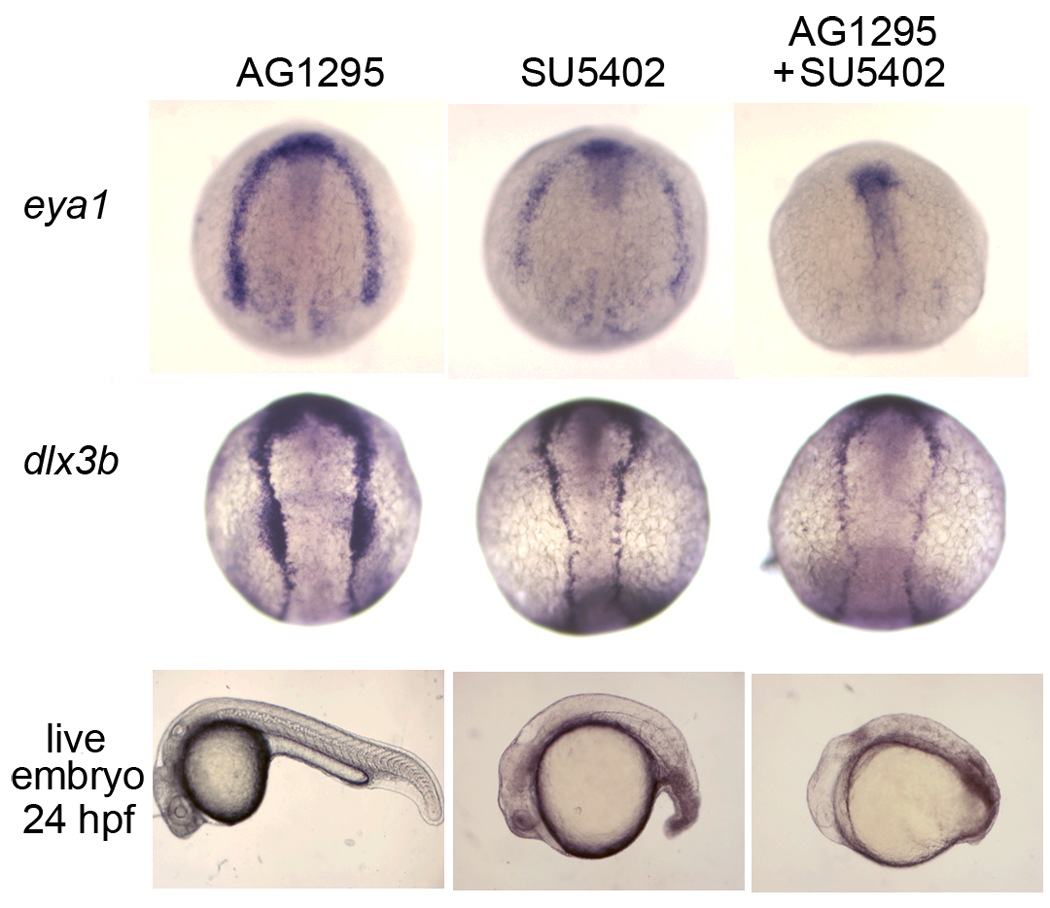

Supplement: Figure S5 — Blocking Fgf and Pdgf signaling leads to downregulation of preplacodal markers. (Upper two rows) Dorsal views showing expression of eya1 and dlx3b at 1l hpf in wild-type embryos that were treated beginning at 8.5 hpf with 15µM AG1295, 25µM SU5402, or both. AG1295 did not cause any significant changes in the expression. SU5402 reduced expression of both genes. Addition of both inhibitors caused loss of eya1 within the preplacodal domain and significant downregulation of dlx3b. (Lower row) Images of live embryos at 24 hpf. Treatment with SU5402 or both SU5402 and AG1295 severely perturbed caudal development and blocked formation of the otic vesicle. (0.96 MB TIF) [file pgen.1001133.s005.tif]

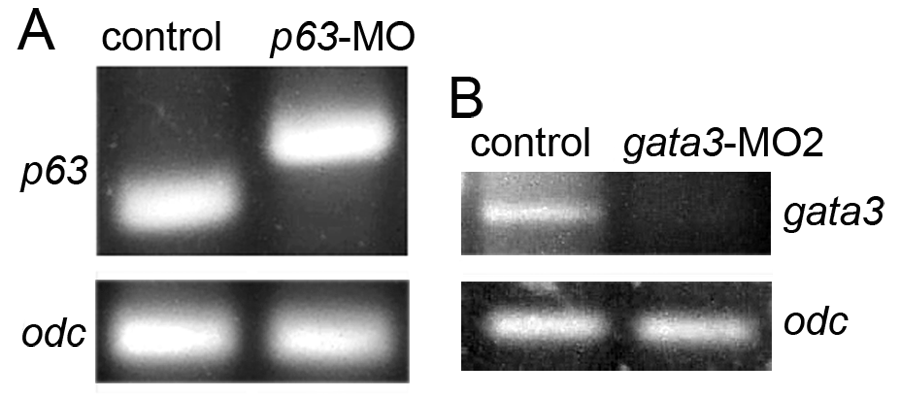

Supplement: Figure S6 — Effects of p63-MO and gata3-MO2 on accumulation of mature mRNA. (A) p63-MO leads to an aberrantly spliced transcript. Control embryos or embryos injected with p63 splice blocker were lysed at 11 hpf to collect mRNA. Primers for p63 and a constitutive control, ornithine decarboxylase (odc) were added to lysates to synthesize cDNA, which was then amplified for 30 cycles. p63-MO caused loss of wild-type transcript and accumulation of an aberrant splice product of higher molecular weight. (B) gata3-MO2 causes loss of gata3 transcript. Control embryos or embryos injected with gata3-MO2 (splice-blocker) were lysed at 12 hpf to collect mRNA. Primers for gata3 and odc were added to lysates to synthesize cDNA, which was then amplified for 30 cycles. Primers for gata3 flanked the splice junction between exons 1 and 2. Primer sequences: gata3: GTGTTGTGTGTATCGGTGAGTG, GAGGAGGAAGAAGCTGGAGG; odc: GGATGTCCTGAAGCACCT, CCCACTGACTGCACGAT; p63: Primers were the same as those used previously [63]. (0.20 MB TIF) [file pgen.1001133.s006.tif]
